# Multiple equilibria enables tunable wetting of droplets on patterned liquid surfaces

**DOI:** 10.1126/sciadv.adw6615

**Published:** 2025-09-19

**Authors:** Xitong Zhang, Hongyu Zhao, Jack R. Panter, Glen McHale, Gary G. Wells, Rodrigo Ledesma-Aguilar, Halim Kusumaatmaja

**Affiliations:** ^1^ Institute for Multiscale Thermofluids, School of Engineering, University of Edinburgh, King’s Buildings, Mayfield Road, Edinburgh EH9 3FD, UK.; ^2^ Department of Physics, Durham University, South Road, Durham DH1 3LE, UK.; ^3^ School of Engineering, University of East Anglia, Norwich, Norfolk NR4 7TJ, UK.

## Abstract

Patterning solid surfaces with varying wettability is important to manage droplets in microfluidics, heat transfer, and printing. Solid surface roughness poses fundamental limitations including contact-line pinning and solid friction. Here, we report an experimental strategy and theoretical design principles for patterned liquid surfaces (PaLS) that combine the controlled wettability from patterning with the ultrasmoothness of a lubricant-infused surface. In contrast to a solid, on PaLS, a droplet can be in 10 different wetting states. This richness arises from the adaptation of the liquid lubricants and can be harnessed to control the apparent contact angle of the droplet over the full range of wettability while removing contact-line pinning effects induced by the solid surface. In the limit of thin liquid films, we derive surface-averaged laws for the apparent contact angle for each wetting state, which capture both experimental and simulation data. Our results provide a distinct approach to surface patterning that exploits the interaction of fluids with lubricant-impregnated surfaces.

## Teaser

Patterning liquid films enables ultrasmooth lubricant-infused surfaces with controlled wettability and multiple droplet states.

## INTRODUCTION

The ability to engineer the interaction of liquid droplets with solid substrates is important in many applications, including microfluidics (*1*, *2*), heat transfer (*3*), inkjet printing (*4*), and surface cleaning (*5*). Patterning a solid substrate, either chemically or topographically, is perhaps the most popular approach to controlling a droplet’s shape, position, and motion. Patterning occurs in many biological systems where droplets come into contact with a solid, and such systems have inspired the design of solid surfaces for specific applications. For example, Namib Desert beetles use patches of hydrophilic and hydrophobic regions of different curvatures that facilitate water harvesting (*6*), and Araucaria leaves have asymmetric structures that result in unidirectional liquid motion (*7*). However, solid surface patterns have a fundamental limitation. Solid surfaces are rough, leading to difficulties in controlling contact-line pinning and strong solid friction forces (*8*, *9*).

Porous or textured solid substrates infused with a liquid lubricant layer, often called slippery liquid-infused porous surfaces (*10*), slippery presuffused surfaces (*11*), or lubricant-impregnated surfaces (*12*), offer the distinct advantage of reducing contact between the droplet and the solid substrate, decreasing contact-line pinning, and enhancing droplet mobility. Such lubricant-infused surfaces also exhibit self-healing, pressure stability, and optical transparency, thus making them attractive in applications such as surface cleaning, anti-icing (*13*, *14*), fog collection (*15*), and heat transfer (*16*, *17*).

The concept of patterned liquid surfaces (PaLS), solid surfaces impregnated by small but distinct domains of two immiscible liquid lubricants, brings together two powerful strategies in surface engineering, combining the benefits of controlled wettability from patterning with the ultrasmoothness of lubricant-infused surfaces. At the same time, PaLS bring to the fore several new fundamental questions in wetting phenomena and multiphase fluid mechanics. On an equivalent patterned solid surface, the configuration of a droplet is determined by a surface average of the individual chemical components of the pattern. In contrast, the adaptability of the liquid lubricants can potentially give rise to new droplet states. On a lubricant-infused surface, a droplet is known to adopt a well-defined apparent contact angle (*18*, *19*), which is determined by the interfacial energies between the solid, the lubricant, the droplet, and the surrounding gas phase (*12*). While some variation of the contact angle can be achieved by choosing different solid/lubricant/droplet combinations (*18*, *20*) or by introducing a topographical micropattern in the solid (*21*, *22*), the range is limited and the resulting surfaces can suffer from contact-line pinning because of contact between the droplet and the solid. Can we overcome these limitations using PaLS?

To address these open challenges, we study liquid droplets and gas bubbles on PaLS. Our experimental method to create the surfaces departs from previous approaches (*23*–*26*) in achieving the coating of a solid substrate with microscale domains of two immiscible lubricants without the need of solid barriers. This creates a small-scale liquid pattern free of solid pinning whose wettability can be adjusted by selecting its composition and geometry. We show that, on PaLS, a liquid droplet or an immersed gas bubble can take 10 distinct wetting states, including complex configurations that depend on whether the lubricants cloak each other and whether the droplet’s surface remains exposed or is cloaked by one or both lubricants. Using a combination of experiments, numerical simulations, and analytical modeling, we demonstrate how choosing the lubricants according to their physical properties can be used to select a specific wetting state, while changing the surface fraction of each lubricant gives us control over the apparent contact angle. The classical Cassie-Baxter law (*27*) fails to capture the apparent contact angle on PaLS. To remedy this failure, we derive a surface-averaged governing law for the apparent contact angle that accounts for the complex lubricant cloaking behaviors on PaLS.

## RESULTS

### Fabrication and visualization of PaLS

**Fig. 1.**
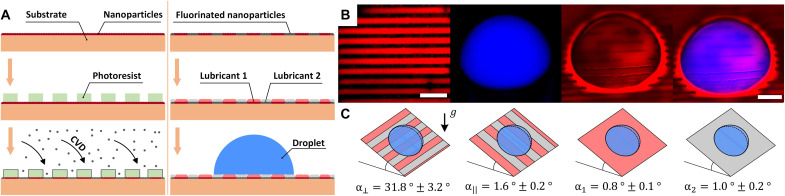
Creating PaLS. (**A**) Surface fabrication procedure. A glass substrate is coated with a thin nanoparticle layer and covered with a photoresist to create an exposed surface pattern. CVD is used to fluorinate the exposed areas, creating a chemical pattern in the nanoparticle layer upon removal of the photoresist. Immiscible liquid lubricants are applied to create a PaLS able to support a droplet of a third liquid in air or a gas bubble surrounded by another liquid. (**B**) Fluorescence microscopy images of a PaLS formed by olive oil/Krytox 50-μm-wide stripes. Olive oil is dyed red, Krytox appears black, and a water droplet is dyed blue. Composite channel images show cloaking of the water droplet by olive oil. Scale bars, 500 μm. (**C**) Sliding angles for a 10-μl water droplet in the perpendicular and parallel directions to the stripes, ${\text{α}}_{⊥}$ and $\alpha_{∥}$, and sliding angles on surfaces coated by olive oil and Krytox only, $\alpha_{1}$ and $\alpha_{2}$, respectively. Data are presented as means ± SD of five measurements.

We developed a robust method to create PaLS by coating a solid substrate with immiscible lubricant domains arranged in a prescribed geometry (see Fig. 1A). Full details of the surface preparation are provided in Materials and Methods. Briefly, precleaned glass slides were coated with Glaco, a silica-based superhydrophobic nanoparticle coating, to create a thin porous layer. A sacrificial layer of photoresist was deposited on the samples using spin coating. The photoresist was etched using photolithography to create the desired pattern. The surfaces were fluorosilanized via chemical vapor deposition (CVD) before the sacrificial photoresist was removed. The resulting nanoparticle layer comprises bare Glaco and fluorosilanized domains. Glaco domains have preferential affinity for alkane-based or siloxane-based lubricants, while fluorosilanized domains have affinity for fluorocarbon lubricants (*20*, *26*). Sequential dip-coating of the surface with suitable lubricant combinations creates a stable surface with well-defined lubricant domains, which, in turn, can support a droplet of a third liquid (or, if desired, a gas bubble surrounded by another liquid). Our method can be used to create domains of arbitrary geometry at high resolution (up to ≈5 μm).

Here, we study the interaction of PaLS with small liquid droplets in air or bubbles in liquids using a parallel-stripe geometry formed by alternating domains of Krytox, a fluorocarbon oil, and either food-grade olive oil or silicone oil (see Fig. 1B). Taking advantage of the high resolution of our surface fabrication method, we vary the width of the stripes between ~5 and 450 μm, which is small compared to the base diameter of the droplet (≈2 mm). The pattern geometry is characterized by the surface fraction of fluorosilanized domains, $f\equiv w_{\text{FS}}/\left(w_{\text{FS}}+w_{G}\right)$, where $w_{G}$ and $w_{\text{FS}}$ are the widths of Glaco and fluorosilanized stripes, respectively.

When placed on the surface, a water droplet adopts a stable equilibrium shape, which depends on the choice of the lubricants and the surface fraction (Fig. 1B). Tilting the surface along the direction of the stripes results in very low sliding angles ($\alpha_{∥}\approx 1{}^{\circ}$), comparable to the sliding angles measured separately on surfaces coated by either lubricant (Fig. 1C), thus indicating minimal pinning from the underlying solid. In sharp contrast, tilting the surface in the transverse direction to the stripes results in much higher sliding angles (e.g., $\alpha_{⊥}\approx 30{}^{\circ}$ for the combination reported in the figure), showing that the lubricant domains introduce a strong anisotropic capillary interaction with the droplet. Details of the sliding angle measurement protocol is provided in the Supplementary Materials.

Figure 1B illustrates the complexity of the interfacial configuration of a droplet on a patterned liquid surface. The droplet (dyed in blue) spreads anisotropically due to the stripe pattern formed by olive oil (dyed in red) and Krytox (undyed, shown in black). Olive oil collects to form a rim around the droplet and cloaks the droplet’s surface. While providing useful information, direct visualization using dyes can be impractical for several reasons. First, droplet cloaking, as is the case shown in the figure, makes it difficult to clearly determine the lubricant configuration underneath the droplet. Second, one needs a third dye to visualize the second lubricant (Krytox). More generally, the dyes must be chosen carefully depending on the liquids used, such that each dye is only miscible in one of the phases and they do not appreciably alter the interfacial tensions. The latter can lead to undesirable changes in the droplet wetting state.

**Fig. 2.**
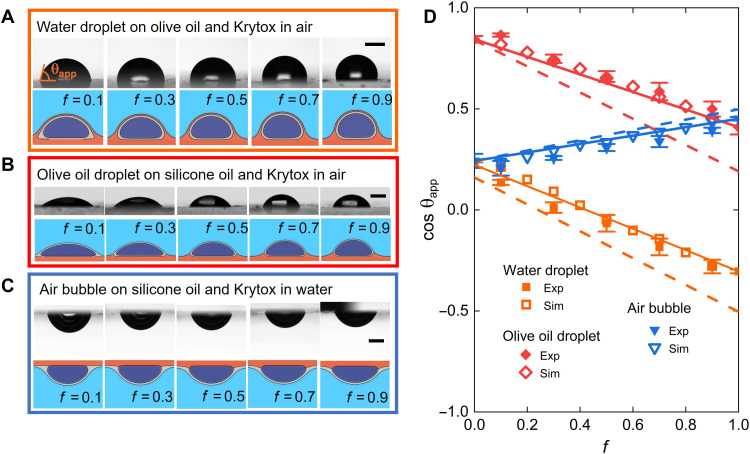
Controlling the wettability of PaLS. (**A** to **C**) Top rows: Experimental images of droplets and bubbles on PaLS composed of stripes of immiscible lubricants at different stripe fraction, *f*. Bottom rows: Cross sections of droplets/bubbles obtained from numerical simulations based on free-energy minimization. (A) Water droplet on a Krytox–olive oil surface surrounded by air. (B) Olive oil droplet on a Krytox–silicone oil surface surrounded by air. (C) Air bubble on a Krytox–silicone oil surface surrounded by water. Scale bars, 1 mm [(A) to (C)]. (**D**) Variation of the cosine of the apparent contact angle with surface fraction. The full and empty markers correspond to experimental (Exp) and simulation (Sim) results, respectively. Experimental data are presented as mean values with error bars representing 1 SD from five independent measurements. The dashed lines correspond to the prediction of the classical Cassie-Baxter law that neglects the effect of both lubricants, while the solid lines are from surface average equations that include the effect of both lubricants.

These observations open several questions. What determines the shape of the droplet? How are the different phases in the system configured in equilibrium? To address these questions, we varied the surface fraction of fluorosilanized stripes, *f*, and studied the shape of the droplet by measuring the apparent contact angle $\theta_{\text{app}}$ (Fig. 2A). In the experiments, the lubricants are in thin films, and so, we define the apparent contact angle by fitting the interface shape to a circular profile whose slope is extrapolated at the level of the flat surface (see the Supplementary Materials). As shown in the figure, increasing the surface fraction has the effect of increasing the apparent angle. We repeated the same experiment for different combinations of the fluids, including situations where the droplet is replaced by an immersed gas bubble. Examples of resulting droplet/bubble configurations are shown in Fig. 2 (B and C) (a full list of the different cases considered in this work is reported in Supplementary Materials). In all cases, the apparent contact angle varies monotonically as the surface fraction changes, suggesting that it is determined by the average effect of the wettability of each lubricant component.

From classical wetting theory on solid substrates, one might expect that the apparent contact angle is governed by Cassie-Baxter law (*27*)

$$\text{cos}\theta_{\text{CB}}=\text{cos}\theta_{1}+f\left(\text{cos}\theta_{2}-\text{cos}\theta_{1}\right)$$ (1)

where the predicted apparent contact angle, $\theta_{\text{CB}}$, results from the spatial average of the contact angles of a droplet observed separately on each lubricant, $\theta_{1}$ and $\theta_{2}$, corresponding to f=0 and f=1. These angles can be measured independently. However, using such “bare” values for $\theta_{1}$ and $\theta_{2}$ in Eq. 1 fails to predict the observed apparent contact angle, as shown by the dashed lines in Fig. 2B. Instead, we find that our data can be captured only if we use $\theta_{1}$ and $\theta_{2}$ that are measured when both lubricants are present (solid lines in the figure), indicating that both lubricants influence the droplet configuration over the full range of surface fractions, even at trace amounts when f→0 and f→1.

### Droplet wetting states

**Fig. 3.**
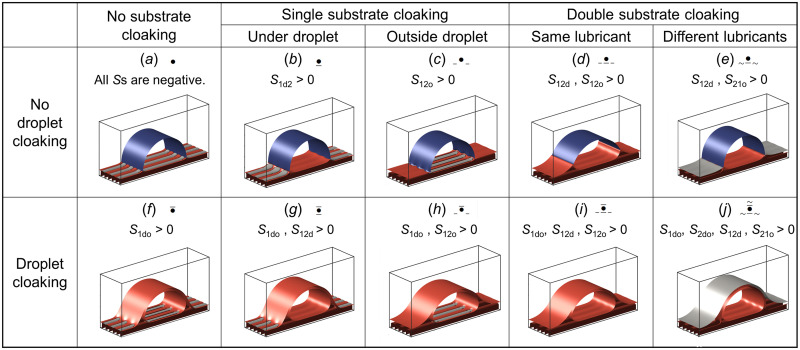
Equilibrium states of droplets on PaLS. The 10 possible equilibrium states can be classified into states where no droplet cloaking occurs [states (*a*) to (*e*)] and where it does [states (*f*) to (*j*)]. Substrate cloaking (where one lubricant cloaks the other) can occur underneath the droplet [(*b*) and (*g*)], outside of it [(*c*) and (*h*)], or in both locations [(*d*) and (*i*)]. Cloaking by different lubricants underneath and outside the droplet can occur in two cases: (*e*) and (*j*). Double cloaking occurs in state (*j*). The images correspond to equilibrium simulation configurations obtained from energy minimization of a four-phase fluid model with f=0.5.

To better understand the lubricant configurations and droplet wetting states, we now develop a theoretical model based on the spreading parameters of the liquids and turn to computer simulations using a phase field method to systematically verify the model predictions and illustrate the possible equilibrium states. A useful quantity to predict the configuration of the four fluid phases in equilibrium is the spreading parameter. Given three different fluid phases (i, j, and k), we define the spreading parameter of fluid i at the interface between fluids j and k as $S_{\text{ijk}}=\gamma_{\text{jk}}-\left(\gamma_{\text{ij}}+\gamma_{\text{ik}}\right)$. Here, $\gamma_{\text{ij}}$ denotes the interfacial tension between fluids i and j. A positive value of $S_{\text{ijk}}$ indicates that a single interface between phases j and k is more energetically expensive than the two interfaces between phases *i-j* and *i-k*. When this happens, it is preferable for fluid i to completely spread at the interface between phases j and k, i.e., we say that fluid i cloaks the *j-k* interface or, in other words, that fluid i cloaks fluid j in the presence of fluid k. We now identify the possible equilibrium states arising from the combination of the cloaking configurations between the lubricants and the droplet. Without loss of generality, we will assume that lubricant 1 (labeled “1”; colored red in Fig. 3) has a larger spreading coefficient than lubricant 2 (“2”; colored gray) at the interface between the droplet (“d”) and outer fluid phase (“o”). In this section, we focus on equilibrium states where the droplet neither completely spreads on nor completely detaches from either lubricant, i.e., $S_{d1o}<0$, $S_{d2o}<0$, $S_{o1d}<0$, and $S_{o2d}<0$ (we shall later discuss situations where these constraints are relaxed). Within these constraints, the lubricants can still cloak the droplet and/or each other. Combinations of the spreading coefficients define 10 unique wetting states, shown in Fig. 3. In terms of classification hierarchy, we first group these states depending on whether cloaking of the droplet occurs. States (*a*) to (*e*) correspond to uncloaked droplet states ($S_{1\text{do}}<0$), while states (*f*) to (*j*) correspond to cloaked droplet states ($S_{1\text{do}}>0$). Within this first classification, one also needs to consider whether, and where, lubricant-lubricant cloaking occurs. For states (*a*) and (*f*), no lubricant-lubricant cloaking occurs. In states (*b*) and (*g*), lubricant cloaking occurs underneath the droplet; whereas in states (*c*) and (*h*), it occurs outside the droplet. In states (*d*) and (*i*), cloaking by the same lubricant occurs both underneath and outside the droplet. However, in states (*e*) and (*j*), different lubricants cloak underneath and outside the droplet: lubricant 1 cloaks lubricant 2 underneath the droplet, and vice versa outside the droplet. In addition, state (*j*) shows double cloaking (*26*), so that a layer of lubricant 1 cloaks the droplet, and this, in turn, is cloaked by lubricant 2.

It is useful to identify the equilibrium states using vignettes, where “•” represents the droplet, and “-” and “∼” are used to indicate cloaking by lubricants 1 and 2 at specific positions. For example, state (*h*), where cloaking by lubricant 1 occurs on the droplet and outside, but not underneath, is symbolized as $\_\bar{•}\_$.

We found that no additional wetting states to those reported in Fig. 3 are possible without raising inconsistencies in the signs of the spreading coefficients. For example, consider a hypothetical state akin to state $\_\bar{•}\_$ but with lubricant 2 (gray) cloaking lubricant 1 (red) underneath the droplet, i.e., $\_\overset{\_}{\underset{˜}{•}}\_$. Since lubricant 2 does not cloak the droplet, we have $S_{2\text{do}}<0$, or $\gamma_{\text{do}}<\gamma_{d2}+\gamma_{2o}$. Furthermore, since lubricant 1 cloaks both the droplet and lubricant 2 outside the droplet, we have $\gamma_{\text{do}}>\gamma_{d1}+\gamma_{1o}$ and $\gamma_{2o}>\gamma_{21}+\gamma_{1o}$. Combining these inequalities yields the condition $\gamma_{d1}<\gamma_{2d}+\gamma_{21}$, or $S_{21d}<0$, which implies that lubricant 2 cannot cloak lubricant 1 underneath the droplet.

To analyze the stability of the wetting states, we carried out numerical simulations of a phase-field model that resolves the interaction between the four fluid phases. Stable states were found by minimizing the free energy

$$E=E_{\text{bulk}}+E_{\text{surf}}+E_{\text{conf}}+E_{\text{ens}}$$ (2)

where $E_{\text{bulk}}$ and $E_{\text{surf}}$ correspond to the bulk and interfacial energy contributions, which allow the coexistence of the four immiscible fluid phases; $E_{\text{conf}}$ represents the energy potential of the pattern, which allows us to confine the lubricants in separate domains; and $E_{\text{ens}}$ takes into account the constant-volume ensemble for the droplet and constant-pressure ensemble for the lubricants. The minimization of *E* was carried out using the Limited-memory Broyden-Fletcher-Goldfarb-Shanno (L-BFGS) algorithm (*28*, *29*), which is routinely used in optimization problems with a large number of degrees of freedom (*30*, *31*). Further details on the free energy model are provided in Materials and Methods.

Snapshots of the droplet configurations obtained from the numerical simulations are shown Fig. 3. We model droplets of cylindrical cross section oriented parallel to the direction of the stripes (*32*). While the model can be used to model full three-dimensional geometries (see the Supplementary Materials for examples), this cylindrical simulation geometry is less expensive computationally. It allows us to capture the effect of the lubricant pattern on the droplet shape and explore a broad range of parameters. The simulations capture the 10 wetting states expected from the theoretical prediction and confirm that no further configurations involving the four fluid phases are possible. The simulations also reveal the presence of lubricant wetting ridges surrounding the droplets, similar to the ridges observed for droplets on surfaces coated with a single lubricant (*33*). However, the interaction with two lubricants introduces undulations of the ridge along the inner and outer edges of the droplet. For example, this effect is particularly noticeable for state $\bar{•}$ in Fig. 3, where one lubricant cloaks the droplet but not the other, either outside or underneath.

Figure 2 also shows the simulation results for the same droplet (or bubble) and lubricant liquids used in the experiments. Figure 2 (A to C) shows the interfacial profiles obtained from the simulations, while the apparent contact angle, and its dependence on the surface pattern fraction, is provided in Fig. 2D. The excellent agreement with experimental data underscores the validity of the simulation approach to capture the effect of PaLS on the droplet configuration. At the same time, the simulations provide detailed information on lubricant cloaking and the corresponding interface shapes, which is difficult to obtain in experiments.

### Apparent contact angle

Our results show that the wettability of PaLS, measured through the apparent contact angle, does not obey the Cassie-Baxter law but follows a surface averaged law that strongly depends on the specific wetting state adopted by the droplet. In this section, we derive a prediction of the apparent contact angle that accounts for the configuration of the full four-phase system. We focus on the regime where the wetting ridge is small compared to the droplet. The experiments show that droplets spread anisotropically on the striped surfaces. Perpendicular to the stripes, the apparent contact angle varies considerably depending on the location of the edge of the droplet relative to the position of the stripes. This is similar to the hysteretic behavior observed for droplets on solid surfaces patterned with chemical stripes and parallel grooves (*34*, *35*). Parallel to the stripes, the apparent contact angle converges to a unique value. This is due to a stronger ability of the droplet edges to spread along the direction of the stripes (as in the configurations shown in Fig. 3).

Close to the droplet edge, the shape of the interface is determined by the balance of interfacial tension forces. For a droplet on a lubricant-infused surface, where there is only one lubricant, these forces correspond to the surface tensions of the interfaces between the lubricant and the outer fluid, the lubricant and the droplet, and the surface of the droplet (*18*, *19*). For PaLS, the corresponding interfaces are between the PaLS and the outer fluid, the PaLS and the droplet, and the droplet’s surface. Each of these interfaces has an effective interfacial tension determined by the cloaking configuration and the surface pattern fraction.

To illustrate this idea, we focus on state (*j*), where the droplet has double cloaking and the lubricants switch cloaking roles underneath and outside of the droplet, i.e., •¯∼¯∼∼. The droplet’s surface comprises three interfaces: the interface between the droplet and lubricant 1 (d1), that between lubricants 1 and 2 (*12*), and that between the outer fluid and lubricant 2 (o2). The effective interfacial tension is thus $\gamma_{\text{do}}^{\text{eff}}=\gamma_{d1}+\gamma_{12}+\gamma_{o2}$. Underneath the droplet, lubricant 1 cloaks lubricant 2. Hence, one has a continuous interface between the droplet and lubricant 1, and a discontinuous interface between lubricants 1 and 2 (because of the pattern). If *f* is the fraction of lubricant 2, then the effective interfacial tension between the droplet and the PaLS is $\gamma_{d,\text{PaLS}}^{\text{eff}}=\gamma_{d1}+f\gamma_{12}$. Following a similar argument, the effective interfacial tension between the outer fluid and the PaLS is $\gamma_{o,\text{PaLS}}^{\text{eff}}=\gamma_{o2}+\left(1-f\right)\gamma_{12}$. Imposing balance between the three interfacial tensions on the plane of the PaLS, we arrive at an analytical law for the apparent contact angle

$$\begin{array}{l} \text{cos}\theta_{\text{app}}=\frac{\gamma_{o,\text{PaLS}}^{\text{eff}}-\gamma_{d,\text{PaLS}}^{\text{eff}}}{\gamma_{\text{do}}^{\text{eff}}}\\ =\frac{\gamma_{o2}-\gamma_{d1}+\left(1-2f\right)\gamma_{12}}{\gamma_{d1}+\gamma_{12}+\gamma_{o2}} \end{array}$$ (3)

The same approach can be used to derive expressions for the apparent contact angle in each wetting state, which we report in Table 1. These expressions demonstrate the complexity and richness of the wettability of the PaLS. For eight of the wetting states, the apparent contact angle depends on the surface pattern fraction, *f*. Notably, for states (*d*) and (*i*), the apparent contact angle is insensitive to *f*. This occurs when the same lubricant cloaks underneath and outside of the droplet, and, hence, the droplet does not interact with the liquid pattern. As a consequence, the apparent contact angle matches that observed for droplets resting on the corresponding single-lubricant surface, making these configurations degenerate in $\theta_{\text{app}}$. One may be tempted to argue that the same should occur for states (*e*) and (*j*) as the droplet only interacts with lubricant 1 underneath and the outer phase only interacts with lubricant 2 outside. However, this disregards the contribution of the interfacial tensions between lubricants 1 and 2, which are weighted differently by the surface pattern fraction in each region.

**Table 1.** Apparent contact angle laws for PaLS. Left: No cloaking. Right: Cloaking. The subscripts in the expressions stand for d = droplet, o = outer fluid, 1 = lubricant 1, and 2 = lubricant 2.

No cloaking		Cloaking	
State ID	$\text{cos}\theta_{\text{app}}$	State ID	$\text{cos}\theta_{\text{app}}$
(*a*) •	$\left[f\left(\gamma_{o1}-\gamma_{d1}\right)+\left(1-f\right)\left(\gamma_{o2}-\gamma_{d2}\right)\right]/\gamma_{\text{do}}$	(*f*) $\bar{•}$	$\left[f\left(\gamma_{1o}-\gamma_{d1}\right)+\left(1-f\right)\left(\gamma_{o2}-\gamma_{d2}\right)\right]/\left(\gamma_{d1}+\gamma_{o1}\right)$
(*b*) $\underline{•}$	$\left[f\gamma_{o1}-\gamma_{d1}+\left(1-f\right)\left(\gamma_{o2}-\gamma_{12}\right)\right]/\gamma_{\text{do}}$	(*g*) $\underline{\bar{•}}$	$\left[f\gamma_{o1}-\gamma_{d1}+\left(1-f\right)\left(\gamma_{2o}-\gamma_{12}\right)\right]/\left(\gamma_{d1}+\gamma_{o1}\right)$
(*c*) _•_	$\left[\gamma_{o1}-f\gamma_{d1}+\left(1-f\right)\left(\gamma_{12}-\gamma_{d2}\right)\right]/\gamma_{\text{do}}$	(*h*) $\_\bar{•}\_$	$\left[\gamma_{o1}-f\gamma_{d1}+\left(1-f\right)\left(\gamma_{12}-\gamma_{d2}\right)\right]/\left(\gamma_{d1}+\gamma_{1o}\right)$
(*d*) $\_\underline{•}\_$	$\left(\gamma_{o1}-\gamma_{d1}\right)/\gamma_{\text{do}}$	(*i*) $\_\overset{¯}{\underset{\_}{•}}\_$	$\left(\gamma_{1o}-\gamma_{d1}\right)/\left(\gamma_{d1}+\gamma_{o1}\right)$
(*e*) •_∼∼	$\left[\gamma_{o2}+\left(2f-1\right)\gamma_{12}-\gamma_{d1}\right]/\gamma_{\text{do}}$	(*j*) •¯∼_∼∼	$\left[\gamma_{o2}-\gamma_{d1}+\left(2f-1\right)\gamma_{12}\right]/\left(\gamma_{d1}+\gamma_{12}+\gamma_{o2}\right)$

Equation 3 can also be used to understand the failure of the classical Cassie-Baxter law, Eq. 1, in describing the wettability of PaLS. For a given wetting state, Eq. 3 can be recast as a surface averaged law, ${\text{cos}\theta_{\text{app}}=\text{cos}\theta_{\text{app}}|}_{f=0}+f\left(\text{cos}\theta_{\text{app}}{|{}_{f=1}-\text{cos}\theta_{\text{app}}|}_{f=0}\right)$. Using state (*j*) as an example yields ${\text{cos}\theta_{\text{app}}|}_{f=0}=\left(\gamma_{o2}-\gamma_{d1}+\gamma_{12}\right)/\left(\gamma_{d1}+\gamma_{12}+\gamma_{o2}\right)$ and ${\text{cos}\theta_{\text{app}}|}_{f=1}=\left(\gamma_{o2}-\gamma_{d1}-\gamma_{12}\right)/\left(\gamma_{d1}+\gamma_{12}+\gamma_{o2}\right)$, showing that the limiting apparent contact angles are affected by both lubricants and not just by one.

### Comparison between theory, simulations, and experiments

To test the prediction of Table 1, we carried out further experiments of liquid droplets and bubbles on PaLS using combinations of water, silicone oil, olive oil, Krytox, and air as working fluids. The choice of these fluids allows us to access five of the 10 possible wetting states, capturing different cloaking configurations. The corresponding spreading coefficients and wetting states are summarized in Table 2. For example, a droplet of olive oil on a patterned liquid surface composed of silicone oil and Krytox surrounded by air [marked “o(sk)a” in the table] has spreading coefficients that map to state (*g*). For each combination, we varied the surface fraction and measured the corresponding apparent contact angle. We also carried out numerical simulations for each wetting state on the same stripe patterns used in the experiments, focusing on controlling the apparent contact angle by either varying the surface fraction or the interfacial tensions.

**Table 2.** Fluid combinations, spreading parameters, and wetting states. The key d(12)o indicates the combination of droplet/bubble fluid (d), the two lubricants (12), and the outer fluid (o). The working fluids used are water (w), Krytox (k), olive oil (o), silicone oil (s), and air (a). Spreading parameters are reported in mN m^−1^. Data are presented as means ± SD of at least five measurements.

d(12)o	$S_{1\text{do}}$	$S_{2\text{do}}$	$S_{1d2}$	$S_{2d1}$	$S_{12o}$	$S_{21o}$	State
w(ok)a	22.5 ± 0.5	2.4 ± 0.5	23.1 ± 0.3	−46.7 ± 0.3	−26.6 ± 0.3	3.0 ± 0.3	(*j*)
o(sk)a	11.1 ± 0.3	2.4 ± 0.5	3.4 ± 0.2	−17.0 ± 0.2	−8.9 ± 0.2	−4.7 ± 0.2	(*g*)
a(sk)w	21.8 ± 0.9	2.4 ± 0.5	−8.9 ± 0.2	−4.7 ± 0.2	14.7 ± 0.8	−28.3 ± 0.8	(*h*)
w(sk)a	21.8 ± 0.9	2.4 ± 0.5	14.7 ± 0.8	−28.3 ± 0.8	−8.9 ± 0.2	−4.7 ± 0.2	(*g*)
s(ko)a	−4.7 ± 0.2	−14.3 ± 0.3	−17.0 ± 0.2	−6.6 ± 0.2	3.0 ± 0.3	−26.6 ± 0.3	(*c*)
a(ko)s	−4.7 ± 0.2	−14.3 ± 0.3	3.0 ± 0.3	−26.6 ± 0.3	−17.0 ± 0.2	−6.6 ± 0.2	(*b*)

**Fig. 4.**
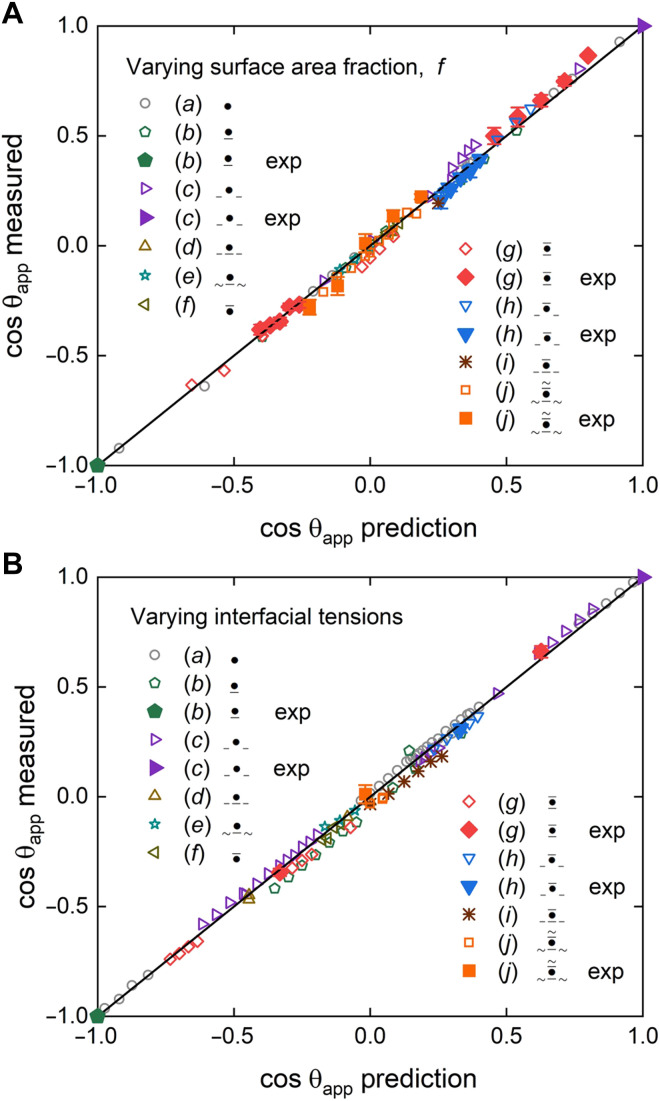
Measured versus predicted apparent contact angles on PaLS. (**A**) Data from experiments and simulations for different fluid combinations and varying surface pattern fraction. (**B**) Data from experiments and simulations at fixed surface fraction f=0.5 and varying interfacial tensions. States and configurations are indicated in the legend. Experimental data are presented as mean values, with error bars indicating 1 SD from five independent measurements.

The results are shown in Fig. 4. The panels show measured versus predicted values of the apparent contact angle for droplets placed on surfaces with different surface pattern fraction, *f* (Fig. 4A), or with different interfacial tensions for a fixed surface fraction f=0.5 (Fig. 4B). The predicted apparent angles are obtained by first determining the spreading coefficients based on the interfacial tensions (which are either measured in the experiments or provided as input parameters in the numerical simulations). This allows us to identify the corresponding wetting state and to select the appropriate expression for $\theta_{\text{app}}$ from Table 2. In total, we tested 190 droplet configurations finding an excellent agreement with the theoretical prediction. Small disparities can be attributed mainly to the fact that the lubricant wetting ridge is small but still finite in size. The wetting ridge can exert forces on the interface of droplets, making the interface curvature adjust to satisfy the balance of Laplace pressure across the interfaces of the ridges and droplets and thus affecting the apparent contact angle (*19*). We discuss the effect of the wetting ridge size on the apparent contact angle in the Supplementary Materials. The results in Fig. 4 underscore the ability to tailor droplet contact angles on PaLS over the full range of wettability (0° to 180°) by varying the lubricant fraction and selecting suitable liquids.

## DISCUSSION

**Fig. 5.**
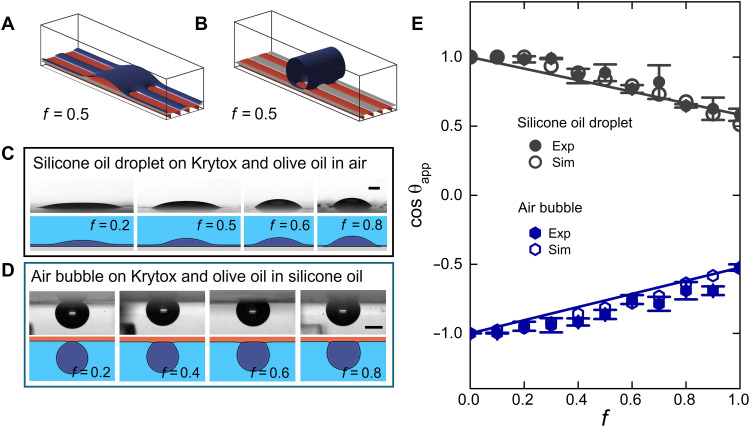
Controlling spreading and dewetting using PaLS. (**A**) A fluid that would otherwise spread into a thin film on a lubricant layer is stabilized into a droplet shape by the second lubricant. (**B**) A fluid that would otherwise detach from a lubricant layer is forced to attach by the second lubricant. (**C** and **D**) Experimental observation of the configurations of [(A) and (B)]. The bottom rows show cross sections of the droplet obtained from numerical simulations. Scale bars, 1 mm [(C) and (D)]. (**E**) Cosine of the apparent contact angle versus surface pattern fraction for the fluid combinations of [(C) and (D)]. Experimental data are presented as mean values, with error bars indicating 1 SD from five independent measurements. The solid line corresponds to the theoretical prediction (see Results).

Here, we have developed a robust method to create PaLS with distinct micrometer-sized lubricant domains. PaLS combine the ultrasmoothness of the liquid lubricants with the structure provided by the surface pattern, thus unlocking an effective mechanism to adjust the wettability of a lubricant-infused surface over the full range of apparent contact angles. We have shown that the lubricant pattern introduces anisotropy in the surface wettability, including capillary adhesion forces that can be used to keep a droplet in place. These forces arise purely from the lubricant domains and can be adjusted by a rational design of the pattern geometry. The richness of multiphase equilibria on PaLS is illustrated by the variety of equilibrium configurations of droplets on these surfaces. Moreover, we have provided a systematic approach to identify and study the equilibrium states based on the spreading coefficients of the specific multiphase fluid system and have developed a rational approach to predict the wettability of the surface via the apparent contact angle. So far, we have explored different possible lubricant cloaking combinations while limiting our study to situations where the droplet shows partial wetting separately on both lubricants. Further equilibrium states are possible if we relax this constraint. For instance, one may consider situations where the droplet either completely spreads on, or completely detaches from, one of the two lubricants. A fluid that completely spreads on lubricant 2 ($S_{d2o}>0$) but not on lubricant 1 ($S_{d1o}<0$) can be stabilized into a droplet shape on a patterned liquid surface as shown in Fig. 5A, thus preventing complete spreading. Likewise, a fluid that completely dewets from lubricant 2 ($S_{o2d}>0$) but not from lubricant 1 ($S_{o1d}<0$) can be forced to attach to the surface using the same approach (Fig. 5B). These situations can be realized experimentally, as shown in Fig. 5 (C and D). Furthermore, because the apparent contact angle is controlled by the wettability of both lubricants, it is possible to vary the apparent contact angle over a broad range by changing the surface pattern fraction. Following the same approach as before, we find

$$\text{cos}\theta_{\text{app}}=\frac{f\left(\gamma_{2o}-\gamma_{2d}\right)+\left(1-f\right)\gamma_{\text{do}}}{\gamma_{\text{do}}}$$

which is in excellent agreement with the experimental and simulation data as presented in Fig. 4E (see table S2 for values of the surface tensions). These results further illustrate how PaLS can be used to control the configuration of droplets and bubbles without the need of direct contact with a solid surface.

In this work, we have focused on PaLS with a stripe-pattern geometry. A natural extension for future work is to consider other surface patterns, including cases where the deformation of the contact line parallel and perpendicular to the liquid domains is more strongly coupled. Therefore, we carried out further simulations of droplets on different patterns, including radial, square, and radial checker-board geometries. The effective contact-angle equations provided in Table 1 remain valid (see the Supplementary Materials).

The ability to control the wettability of PaLS down to the scale of micrometer-sized domains offers advantages in practical applications. For example, in Micro Electro-Mechanical Systems (MEMS) and droplet microfluidics technologies, interactions between droplets and solids are often addressed using chemical or topographical patterns, both of which are limited by contact-line pinning (*1*, *2*). On PaLS, pinning on the solid is eliminated while the structure of the lubricant domains introduces capillary interactions, which can be engineered to control the position of a target droplet by creating anchoring points or to induce self-propulsion via a gradient in the pattern geometry. In inkjet printing applications (*4*), droplet-droplet interactions mediated by the surface are crucial but are often limited by the quality of the underlying solid substrate. Here, PaLS could be used as a primer layer (*36*) and exploited to increase the resolution quality of current wet-on-wet printing techniques (*37*).

## MATERIALS AND METHODS

### Surface preparation

Precleaned glass slides (CLS294775X25-72EA, Corning) were dip-coated in a silica-based nanoparticle suspension (Glaco Mirror Coat Zero, SOFT99 Corp.) at a speed of 1 mm s^−1^ using a robot (Ossila Ltd.). A residence time of 30 s was allowed before the substrates were withdrawn from the solution at a speed of 0.5 mm s^−1^. The substrates were left to dry for 3 min. The dip-coating process was repeated five times to ensure that a uniform layer of nanoparticles was formed on the surfaces (*38*, *39*).

The substrates were primed with hexamethyldisilazane vapor for 10 min. A sacrificial layer of photoresist (SPR350, Megaposit) was deposited on the substrates using a spin coater (POLOS200, SPS POLOS) at a speed of 4000 rpm. The coated substrates were baked at 90°C for 90 s. The photoresist was exposed to ultraviolet light at 44 mJ cm^−2^ to create the desired pattern using a microwriter (ML3, Durham Magneto Optics). The substrates were rinsed with a developer solution (MF-26A, Kayaku Advanced Materials) to remove the exposed regions of the photoresist. The substrates were treated with oxygen plasma at 200 W for 2 min. The surfaces were then fluorosilanized via CVD using trichloro(1H,1H,2H,2H-perfluorooctyl)silane (no. 448931, Sigma-Aldrich) before the sacrificial photoresist was removed by rinsing with isopropanol (propan-2-ol, ≥99.8%, Sigma-Aldrich) for 30 s. The substrates were coated with the target lubricants using sequential dip-coating at a withdrawal speed of 0.01 mm s^−1^.

### Simulation model

To determine the stable wetting states of droplets on PaLS, numerical simulations of a phase-field model were conducted by minimizing the total free energy of the four fluid phases, as given in Eq. 2. Here, we describe the different contributions to the free energy. The simulation setup is discussed in the Supplementary Materials. The bulk free energy $E_{\text{bulk}}$ is defined as

$$\begin{array}{cc} E_{\text{bulk}}= & \int \left\{\sum_{i=1}^{N-1}\sum_{j=i+1}^{N}k_{\text{ij}}\left[f\left(c_{i}\right)+f\left(c_{j}\right)+f\left(c_{i}+c_{j}\right)\right]\right.\\ & \left.+\lambda \sum_{i=1}^{N-2}\sum_{j=i+1}^{N-1}\sum_{k=j+1}^{N}c_{i}^{2}c_{j}^{2}c_{k}^{2}\right\}\mathrm{dV} \end{array}$$ (4)

in which N=4 is the total number of the fluid phases and *V* is the total volume occupied by them. The first term allows for the coexistence of the four fluid phases. Here, the local concentration of the i-th fluid phase, $c_{i}$, can be regarded as an order parameter, which varies from 0 to 1. The function $f\left(c_{i}\right)=c_{i}^{2}{\left(c_{i}-1\right)}^{2}$ is a double-well potential whose minima, located at $c_{i}=0$ and $c_{i}=1$, characterize the pure phases of the mixture. $k_{\text{ij}}$ are numerical prefactors that are related to the interfacial tensions (see Eq. 6). Following Boyer and Lapuerta (*40*), the second term in Eq. 4 is introduced to avoid the instability of the phase field when one phase spreads over another. In this term, λ is a positive stabilization parameter, which we set to λ=50 in lattice unit (l.u.).

The interfacial free energy $E_{\text{surf}}$ is defined by

$$\begin{array}{cc} E_{\text{surf}}= & \int \left[\sum_{i=1}^{N-1}\frac{k_{\text{ii}}^{\prime }}{2}{|\nabla c_{i}|}^{2}+\right.\\ & \left.\sum_{i=1}^{N-2}\sum_{j=i+1}^{N-1}\frac{k_{\text{ij}}^{\prime }}{2}{|\nabla \left(c_{i}+c_{j}\right)|}^{2}\right]\mathrm{dV} \end{array}$$ (5)

In equilibrium, it is possible to derive expressions for the interfacial tensions between the different phases, $\gamma_{\text{ij}}$, from $E_{\text{bulk}}$ and $E_{\text{surf}}$. They are related to the *k* prefactors by

$$\gamma_{\text{ij}}=\frac{\alpha }{3}\sum_{m=1}^{N-1}\sum_{n=m+1}^{N}w_{\text{mn}}k_{\text{mn}}$$ (6)

where the weights $w_{\text{mn}}$ are defined by

$$w_{\text{mn}}=\left\{\begin{array}{cc} 1 & \text{if}\;m=i\;\text{or}\;j\;\text{for}\;\text{any}\;n\\ 1 & \text{if}\;n=i\;\text{or}\;j\;\text{for}\;\text{any}\;m\\ 0 & \text{if otherwise} \end{array}\right.$$ (7)

and α is a measure of the width of diffuse interface, set to be 1.0 l.u. The parameters $k_{\text{ij}}^{\prime }$ are associated with the spreading parameters $S_{N\text{ij}}$ via

$$k_{\mathrm{ij}}^{\prime }=\left\{\begin{array}{cc} -3\alpha S_{N\text{ij}} & \text{if}\;i\neq j\\ 3\alpha \left(2\gamma_{iN}+\sum_{m=1,m\neq i}^{N-1}S_{N\text{im}}\right), & \text{if}\;i=j \end{array}\right.$$ (8)

In our simulations, the surface tension parameters are chosen to mimic the liquids used in the experiments (see table S2), or they are systematically varied to access different wetting states. In the experiments, the micro–surface patterns make one set of regions of the solid substrate energetically attractive to the first lubricant liquid, and the other regions attractive to the second lubricant liquid. To model this spatial confinement of each lubricant liquid phase in the simulations, we use the confining free energy

$$E_{\text{conf}}=\int_{\Omega_{\text{lub}}}\Psi_{\text{conf}}\mathrm{dV}$$ (9)

where $\Omega_{\text{lub}}$ represents the lubricant region with a thickness of 10 l.u. from the bottom of the simulation domain. Without loss of generality, we now index the two lubricants as phases 1 and 2, the droplet as phase d, and the surrounding fluid as phase o. In regions of the space where lubricant 1 is confined, $\Psi_{\text{conf}}$ takes the form

$$\Psi_{\text{conf}}=C_{\text{conf}}\left[{\left(c_{d}-1\right)}^{3}\left(3c_{d}+1\right)+c_{1}^{3}\left(3c_{1}-4\right)\right]$$ (10)

whereas in regions of space where lubricant 2 is confined

$$\Psi_{\text{conf}}=C_{\text{conf}}\left[{\left(c_{d}-1\right)}^{3}\left(3c_{d}+1\right)+c_{2}^{3}\left(3c_{2}-4\right)\right]$$ (11)

where $C_{\text{conf}}$ is the confining potential strength, set at $C_{\text{conf}}=1$ in lattice unit. The first terms in both Eqs. 10 and 11 make the regions of space where the lubricant is confined repulsive to the droplet phase. The second terms in each equation make the region attractive to lubricants 1 and 2, respectively.

The ensemble free energy, $E_{\text{ens}}=E_{\text{drop}}+E_{\text{lub}}$, accounts for the constant-volume ensemble for the droplet and constant-pressure ensemble for the lubricants. The constant volume ensemble for the droplet is enforced by the term

$$E_{\text{drop}}=k_{\text{vol}}{\left(V_{\text{drop}}-V_{0}\right)}^{2}$$ (12)

Here, $k_{\text{vol}}=0.001$ (in lattice unit) is a constant prefactor controlling the strength of the constraint, $V_{\text{drop}}$ is the measured volume of droplet phase in the domain, and $V_{0}$ is the target volume of the droplet. For the lubricant liquids, we apply the pressure constraint (instead of imposing a constraint on their volume) to ensure that a sufficient amount of lubricants is available to fully spread on the droplet and/or the other interfaces. The pressure constraint is expressed as follows

$$E_{\text{lub}}=-p_{0}V_{\text{lub}}$$ (13)

where $p_{0}<0$ is the pressure of the lubricant liquids. We use a range of $-0.05<p_{0}<-0.01$ in this work. A small absolute value of $p_{0}$ indicates less work to increase the volume of the lubricant layer at the expense of additional deformation of its interface. This leads to larger ridge sizes, especially in the cases where one lubricant fully wets the surface of the lubricant layer outside of the droplet and also cloaks the droplet interface.

## Supplementary Materials

### This PDF file includes:

Supplementary Text S1 to S7 Figs. S1 to S4 Tables S1 to S2 References
