# Supplementary material for: Multiple equilibria enables tunable wetting of droplets on patterned liquid surfaces
Source: Sci Adv. 2025 Sep 19;11(38):eadw6615. doi: 10.1126/sciadv.adw6615 (PMC12448060; doi:10.1126/sciadv.adw6615)
Supplement: Supplementary file 1 — Supplementary Text S1 to S7 Figs. S1 to S4 Tables S1 to S2 References [file sciadv.adw6615_sm.pdf]

Science Advances
AAAS

Supplementary Materials for

Multiple equilibria enables tunable wetting of droplets on patterned liquid surfaces

Xitong Zhang *et al.*

Corresponding author: Glen McHale, glen.mchale@ed.ac.uk; Gary G. Wells, gary.wells@ed.ac.uk; Rodrigo Ledesma-Aguilar, rodrigo.ledesma@ed.ac.uk; Halim Kusumaatmaja, halim.kusumaatmaja@ed.ac.uk

Sci. Adv. **11**, eadw6615 (2025)
DOI: 10.1126/sciadv.adw6615

This PDF file includes:

Supplementary Text S1 to S7
Figs. S1 to S4
Tables S1 to S2
References

Supplementary Text S1

Interfacial tension measurements

Interfacial tensions were measured using the pendant droplet method. A droplet (fluid i) was dosed using a syringe pump into the surrounding phase (fluid j) in a transparent cuvette container. The droplet was recorded by a CCD camera and its shape was processed by ImageJ using a pendant droplet analysis algorithm (*41*). The interfacial tension, γ_{ik}, is obtained from the balance between the capillary and gravitational forces,

$$\gamma_{\mathrm{ij}} = \frac{\Delta\rho_{\mathrm{ij}} g R}{\beta}, \tag{S1}$$

where $\Delta\rho_{\mathrm{ij}}$ is the density difference between the fluids, g is the acceleration due to gravity, R is the radius of curvature at the tip of the droplet and β is a factor derived from the shape of the droplet. For each fluid-fluid pair, the interfacial tension was determined by averaging five independent measurements. The standard error was obtained by the error propagation based on Eq. S1. The measured values for each fluid-fluid pair are reported in Table S1.

Supplementary Text S2

Apparent contact angle measurements

The apparent contact angle of a droplet in contact with a lubricant-infused surface is affected by the presence of the wetting ridge formed by the lubricant and the droplet *(19,42)*. We consider two natural geometric choices to define the apparent contact angle: the first is determined by the slope of the droplet–gas interface at the intersection between the wetting ridge and the droplet, denoted θ^*_{app}; the second is determined by the slope of the virtual droplet–gas interface extrapolated to the position of the surface of the lubricant layer, denoted θ_{app}. When there is little excess lubricant and the wetting ridge is small, as is the case in the experiments, we expect that there is no significant difference between θ^*_{app} and θ_{app}. Therefore, we measure the apparent contact angle by fitting the interface profile of the droplet to a third-degree polynomial and extrapolating its slope to the baseline of the surface. Measurements were repeated five times and averaged. The error is reported as the standard deviation of the sample.

Supplementary Text S3

Sliding angle measurements

A sample PaLS made of Krytox and olive oil stripes was placed on a platform connected to a rotating stage (Stepper Motor Rotation Mount, Thorlabs). The initial orientation of the stage was parallel to the horizontal. A 10-μL droplet was gently placed on the PaLS and allowed to equilibrate. The surface was then tilted at a speed of $0.02^\circ\ \mathrm{s}^{-1}$. The tilting angle at which the contact line of the droplet started to move was recorded as the sliding angle. Due to the anisotropy of the stripe pattern, the experiment was carried out by tilting the surface along the parrallel and the perpendicular directions to the stripes separately. The sliding angle for a droplet on the corresponding single-lubricant infused surface was measured for comparison. The standard deviation was given from the measurement of four independent experiments.

Supplementary Text S4

Cylindrical droplet simulation

In this work we primarily simulate cylindrical droplets on PaLS since we are interested in the apparent contact angle parallel to the stripes. Figure S1 shows a schematic of the computational simulation domain. The simulation box has a rectangular cross-section of dimensions $L_{\mathrm{y}} = 200$ and $L_{\mathrm{z}} = 100$, both in lattice units (l.u.). The width of the domain, L_{x}, is fixed depending on the width of the lubricant stripes. A minimum of three periods of the lubricant pattern are resolved in the simulations, with a minimum stripe width of 10 l.u. The top and bottom boundaries of the domain are modeled as solid walls, while mirror boundary conditions are applied for the remaining boundaries. The lubricant layer is initially subjected to a pressure constraint and undergoes a free energy minimization process. Following this step, the droplet is initialized as a half-cylinder with a constant volume constraint. We typically set the droplet volume to be at least 12% of the volume of the domain. Subsequently, we minimise the free energy of the entire system.

Table S2 lists the interfacial tensions used in the simulations that are compared with the corresponding experimental cases in Figures 2, 4, and 5 of the main paper. In the simulations, the results depend on the ratio of the interfacial tensions, rather than their absolute values.

Supplementary Text S5

3D simulation of a spherical droplet on stripe patterns

We conducted a series of full 3D simulations for a water droplet resting on striped domains of Krytox and olive oil, surrounded by air as the ambient fluid. In this set of simulations, the droplet is initialized as a spherical droplet which then relaxes to its equilibrium configuration. The droplet shape is elongated parallel to the stripes and the apparent contact angle is measured parallel to the stripes. Figure S2A shows the apparent contact angles obtained from the cylindrical and spherical droplet simulation setups. Both agree well with those from the experimental measurements and the theory presented in the main paper. This confirms that the cylindrical droplet setup is adequate to capture the apparent contact angle of a droplet on PaLS. Figure S2B presents snapshots of the central cross-section from the full 3D simulations when the Krytox area fraction is $f = 0.3$ and 0.7. The double-layer cloaking of the droplet corresponds to state (j). Figure S2C shows the 3D droplet morphologies when $f = 0.3$ and 0.7.

Supplementary Text S6

3D simulations of spherical droplets on other patterns

Beyond stripe patterns, we also perform full 3D simulations to examine the wetting behaviour of olive oil droplets on PaLS substrates with three different patterns: radial, square, and radial-checkerboard configurations, see Figure S3. The lubricants are Krytox and silicone oil, and the ambient fluid is air. This places the system in wetting state (g). In all three cases, the droplet is initially placed at the centre of the patterned surface, and the droplet volume is quasi-statically reduced from 0.22 V_{domain} to 0.05 V_{domain}, where V_{domain} is the total volume of the computational domain. The computational domain measures $L_{\text{x}} \times L_{\text{y}} \times L_{\text{z}} = 320 \times 320 \times 90\,\text{l.u.}^3$. The change in droplet volume allows the contact line to cross multiple pattern repeats, as can be observed in Figure S3B, and we can compare the measured apparent contact angles with the model prediction for state (g) in the main text, see Figure S3A.

For the radial pattern, each Krytox sector spans a central angle of $12°$, leading to a Krytox area fraction of $f = 0.27$. The measured apparent contact angles are essentially constant (varying over a very narrow range of $42.9°$ and $43.7°$), in full agreement with the predicted value of $43.3°$.

In the square pattern, the side length of a Krytox square domain is fixed at 10 l.u., leading to an area fraction of $f = 0.25$. For the radial checkerboard pattern, which has a Krytox area fraction of $f = 0.50$, we introduce 16 radial sectors with a width of $22.5°$, and the radial cell width is set to 15 l.u.. In both cases, the repeat units of the patterns are considerably smaller than the droplet size, and hence the droplet shape remains close to being isotropic. The measured apparent contact angles oscillate around the predicted theoretical values: $40.4°$ - $44.8°$ ($42.7°$ in theory) for the square pattern, and $46.8°$ - $57.1°$ ($51.2°$ in theory) for the radial checkerboard.

In Figure S3B, we can also observe directional capillary interactions. The droplet contact lines spread along the boundaries of square and radial checkerboard patterns. This is reminiscent of the experimental observation in Figure1C of the main text when tilting the surface in the transverse direction to the stripes. The discontinuities in the contact angles (most clearly seen for the radial checkerboard pattern) are due to the droplet contact lines receding quickly across the more hydrophobic Krytox domains as the droplet volume is reduced.

Supplementary Text S7

Effect of lubricant ridge size on the apparent contact angle

We carried out tests to characterize the effect of the size of the lubricant ridge on the apparent contact angle of the droplet. For a given patterned liquid surface, we varied the pressure constraint as a means to vary the size of the lubricant ridge. We measured the apparent contact angles θ_{app} and θ^*_{app} using the definitions given in section Supplementary Text S1. As shown in Figure S4A, θ^*_{app} increases with decreasing ridge size, h, defined as the height of the intersection of the ridge with the droplet surface. On the other hand, θ_{app} does not vary significantly with ridge size. Figure S4B shows the difference between θ_{app} and θ^*_{app} vs h/H, where H is the height of the droplet. We found that θ^*_{app} approaches θ_{app} and the fitted lines converge to zero as h/H decreases. The highlighted zone represents the range of ridge height observed in our experiments. Figure S4C shows the comparison of these simulation data with the theoretical prediction. In all the cases shown here, the simulation data for θ_{app} are in excellent agreement with the theoretical prediction.

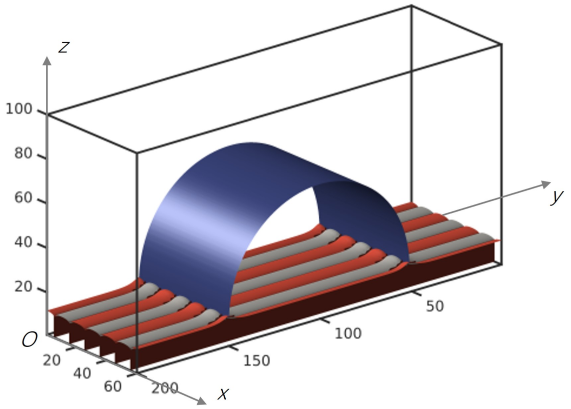

Figure S1: **Sketch of computational domain in simulation.** For clear observation purposes, only the surface of the droplet is shown, and the interior of the droplet is set to a transparent color.

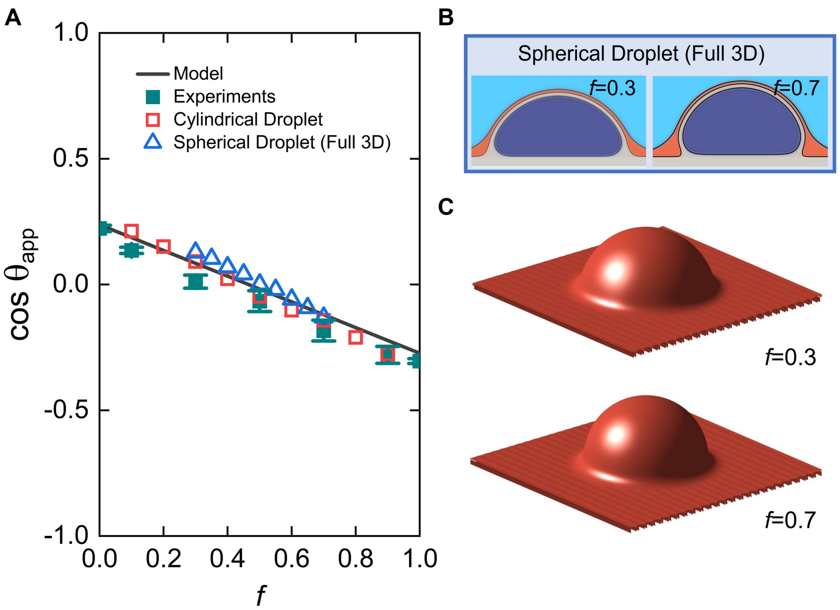

Figure S2: **Comparison of cylindrical (2-D) and spherical (3-D) droplet simulations with experimental data for a water droplet on an olive-oil/Krytox PaLS in air. A** Apparent contact angles from both simulations and experiments versus Krytox area fraction f; symbols show simulations and experiments, the solid line shows the model prediction. Experimental data are presented as mean values, with error bars indicating one standard deviation from five independent measurements. **B** Cross-sectional snapshots from the 3-D simulation at $f = 0.3$ and $f = 0.7$; Krytox is shown in orange and olive oil in grey. **C** Equilibrium droplet shapes from the 3-D simulation for the same two area fractions.

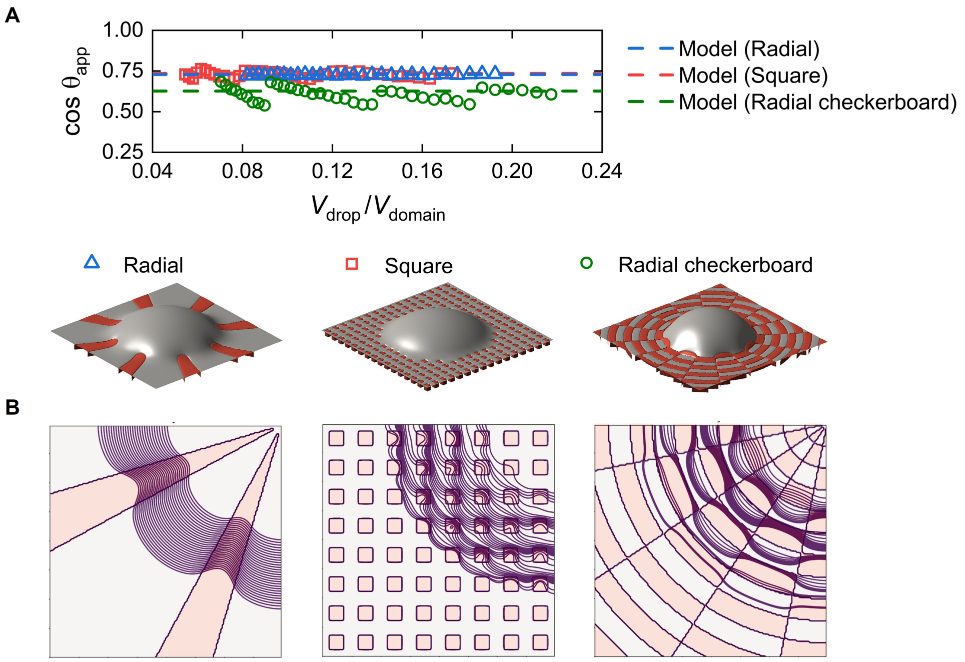

Figure S3: **Wettability of radial, square and radial checkerboard patterned PaLS. A** First row: variation of the cosine of the apparent contact angle on droplet volume. Droplet volume is non-dimensionalized by the volume of the whole computational domain. Dashed lines are model predictions considering lubricant cloaking. Second row: configurations of representative droplets on each pattern; red and grey denote Krytox and silicone oil, respectively. **B** Contours of droplet–PaLS contact lines for selected droplet volumes on the three patterns. For clarity, only one-quarter of the domain is displayed. Light red indicates Krytox, and light grey indicates silicone oil.

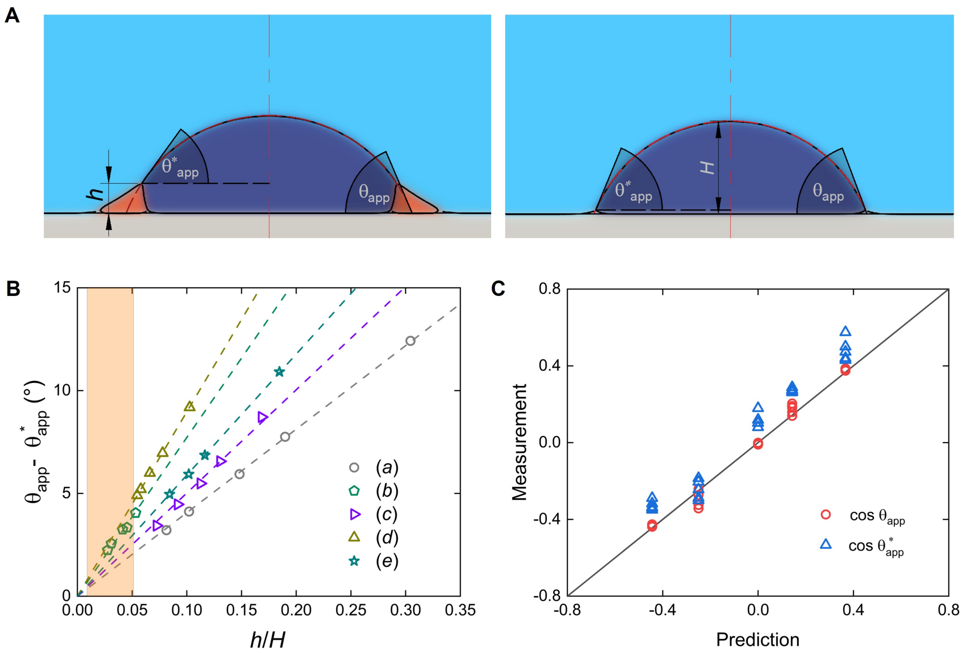

Figure S4: Variation of the apparent contact angle with lubricant ridge size. **A** Schematic illustrating the variation of θ^*_{app} and θ_{app} with the lubricant ridge size. **B** Difference between θ_{app} and θ^*_{app} as a function of h/H. The highlighted zone represents the range of h/H observed in the experiments. **C** Comparison of θ^*_{app} and θ_{app} from **B** with theoretical predictions.

Table S1: **Measured interfacial tensions between the fluids used in the experiments ($\mathbf{mN\,m^{-1}}$).** Subscripts denote water (w), Krytox (k), olive oil (o), silicone oil (s), and air (a). Values are reported as mean ± standard deviation, based on at least five independent measurements.

γ_{wk}	γ_{wo}	γ_{ws}	γ_{wa}	γ_{ko}	γ_{ks}	γ_{ka}	γ_{os}	γ_{oa}	γ_{sa}
52.5 ± 0.2	17.6 ± 0.2	30.0 ± 0.5	71.7 ± 0.4	11.8 ± 0.1	6.8 ± 0.1	16.8 ± 0.1	1.6 ± 0.1	31.6 ± 0.3	18.9 ± 0.1

Table S2: **Interfacial tensions used in simulations for the corresponding experimental cases.** The interfacial tensions are in lattice units (l.u.). The key d(12)o indicates the combination of droplet/bubble fluid (d), the two lubricants (1 and 2), and the outer fluid (o). The working fluids used are water (w), Krytox (k), olive oil (o), silicone oil (s), and air (a).

d(12)o	γ_{d1}	γ_{d2}	γ_{do}	γ_{12}	γ_{1o}	γ_{2o}	Wetting state
w(ok)a	1.780	0.596	2.432	0.400	0.568	1.072	(j)
o(sk)a	0.200	1.400	2.900	0.800	2.350	2.050	(g)
a(sk)w	0.641	0.569	2.432	0.231	1.050	1.780	(h)
w(sk)a	1.050	1.780	2.432	0.231	0.641	0.569	(g)
s(ko)a	0.360	0.085	1.000	0.624	0.889	1.600	(c)
a(ko)s	0.889	1.600	1.000	0.624	0.360	0.085	(b)

REFERENCES AND NOTES

1. R. Malinowski, I. P. Parkin, G. Volpe, Advances towards programmable droplet transport on solid surfaces and its applications. *Chem. Soc. Rev.* **49**, 7879–7892 (2020).

2. Q. Zeng, B. Wang, Z. Guo, Recent advances in microfluidics by tuning wetting behaviors. *Mater. Today Phys.* **40**, 101324 (2024).

3. A. Tokunaga, T. Tsuruta, Enhancement of condensation heat transfer on a microstructured surface with wettability gradient. *Int. J. Heat Mass Transf.* **156**, 119839 (2020).

4. D. Lohse, Fundamental fluid dynamics challenges in inkjet printing. *Annu. Rev. Fluid Mech.* **54**, 349–382 (2022).

5. A. J. Mazaltarim, J. J. Bowen, J. M. Taylor, S. A. Morin, Dynamic manipulation of droplets using mechanically tunable microtextured chemical gradients. *Nat. Commun.* **12**, 3114 (2021).

6. A. R. Parker, C. R. Lawrence, Water capture by a desert beetle. *Nature* **414**, 33–34 (2001).

7. S. Feng, P. Zhu, H. Zheng, H. Zhan, C. Chen, J. Li, L. Wang, X. Yao, Y. Liu, Z. Wang, Three-dimensional capillary ratchet-induced liquid directional steering. *Science* **373**, 1344–1348 (2021).

8. D. Quéré, Wetting and roughness. *Annu. Rev. Mat. Res.* **38**, 71–99 (2008).

9. D. Bonn, J. Eggers, J. Indekeu, J. Meunier, E. Rolley, Wetting and spreading. *Rev. Mod. Phys.* **81**, 739–805 (2009).

10. T.-S. Wong, S. H. Kang, S. K. Y. Tang, E. J. Smythe, B. D. Hatton, A. Grinthal, J. Aizenberg, Bioinspired self-repairing slippery surfaces with pressure-stable omniphobicity. *Nature* **477**, 443–447 (2011).

11. A. Lafuma, D. Quéré, Slippery pre-suffused surfaces. *Europhys. Lett.* **96**, 56001 (2011).

12. J. D. Smith, R. Dhiman, S. Anand, E. Reza-Graduno, R. E. Cohen, G. H. McKinley, K. K. Varanasi, Droplet mobility on lubricant-impregnated surfaces. *Soft Matter* **9**, 1772–1780 (2013).

13. P. Kim, T.-S. Wong, J. Alvarenga, M. J. Kreder, W. E. Adorno-Martinez, J. Aizenberg, Liquid-infused nanostructured surfaces with extreme anti-ice and anti-frost performance. *ACS Nano* **6**, 6569–6577 (2012).

14. P. W. Wilson, W. Lu, H. Xu, P. Kim, M. J. Kreder, J. Alvarenga, J. Aizenberg, Inhibition of ice nucleation by slippery liquid-infused porous surfaces (SLIPS). *Phys. Chem. Chem. Phys.* **15**, 581–585 (2013).

15. B. Qi, X. Yang, X. Wang, Ultraslippery/hydrophilic patterned surfaces for efficient fog harvest. *Colloids Surf. A Physicochem. Eng. Asp.* **640**, 128398 (2022).

16. S. Anand, A. T. Paxson, R. Dhiman, J. D. Smith, K. K. Varanasi, Enhanced condensation on lubricant-impregnated nanotextured surfaces. *ACS Nano* **6**, 10122–10129 (2012).

17. R. Xiao, N. Miljkovic, R. Enright, E. N. Wang, Immersion condensation on oil-infused heterogeneous surfaces for enhanced heat transfer. *Sci. Rep.* **3**, 1988 (2013).

18. G. McHale, B. V. Orme, G. G. Wells, R. Ledesma-Aguilar, Apparent contact angles on lubricant-impregnated surfaces/SLIPS: From superhydrophobicity to electrowetting. *Langmuir* **35**, 4197–4204 (2019).

19. C. Semprebon, G. McHale, H. Kusumaatmaja, Apparent contact angle and contact angle hysteresis on liquid infused surfaces. *Soft Matter* **13**, 101–110 (2017).

20. G. McHale, N. Afify, S. Armstrong, G. G. Wells, R. Ledesma-Aguilar, The liquid young's law on slips: Liquid–liquid interfacial tensions and zisman plots. *Langmuir* **38**, 10032–10042 (2022).

21. M. S. Sadullah, G. Launay, J. Parle, R. Ledesma-Aguilar, Y. Gizaw, G. McHale, G. G. Wells, H. Kusumaatmaja, Bidirectional motion of droplets on gradient liquid infused surfaces. *Commun. Phys.* **3**, 166 (2020).

22. G. Launay, M. S. Sadullah, G. McHale, R. Ledesma-Aguilar, H. Kusumaatmaja, G. G. Wells, Self-propelled droplet transport on shaped-liquid surfaces. *Sci. Rep.* **10**, 14987 (2020).

23. N. Vogel, R. A. Belisle, B. Hatton, T.-S. Wong, J. Aizenberg, Transparency and damage tolerance of patternable omniphobic lubricated surfaces based on inverse colloidal monolayers. *Nat. Commun.* **4**, 2176 (2013).

24. E. Ueda, P. A. Levkin, Micropatterning hydrophobic liquid on a porous polymer surface for long-term selective cell-repellency. *Adv. Healthc. Mater.* **2**, 1425–1429 (2013).

25. D. Paulssen, S. Hardt, P. A. Levkin, Droplet sorting and manipulation on patterned two-phase slippery lubricant-infused surface. *ACS Appl. Mater. Interfaces* **11**, 16130–16138 (2019).

26. M. Pelizzari, G. McHale, S. Armstrong, H. Zhao, R. Ledesma-Aguilar, G. G. Wells, H. Kusumaatmaja, Droplet self-propulsion on slippery liquid-infused surfaces with dual-lubricant wedge-shaped wettability patterns. *Langmuir* **39**, 15676–15689 (2023).

27. A. B. D. Cassie, S. Baxter, Wettability of porous surfaces. *Trans. Faraday Soc.* **40**, 546–551 (1944).

28. D. C. Liu, J. Nocedal, On the limited memory BFGS method for large scale optimization. *Math. Program.* **45**, 503–528 (1989).

29. J. Nocedal, Updating quasi-newton matrices with limited storage. *Math. Comput.* **35**, 773–782 (1980).

30. J. R. Panter, J. Chen, T. Zhang, H. Kusumaatmaja, Harnessing energy landscape exploration to control the buckling of cylindrical shells. *Commun. Phys.* **2**, 151 (2019).

31. Y. Li, S. J. Avis, J. Chen, G. Wu, T. Zhang, H. Kusumaatmaja, X. Wang, Reconfiguration of multistable 3D ferromagnetic mesostructures guided by energy landscape surveys. *Extreme Mech. Lett.* **48**, 101428 (2021).

32. B. M. Mognetti, H. Kusumaatmaja, J. Yeomans, Drop dynamics on hydrophobic and superhydrophobic surfaces. *Faraday Discuss.* **146**, 153–165 (2010).

33. F. Schellenberger, J. Xie, N. Encinas, A. Hardy, M. Klapper, P. Papadopoulos, H.-J. Butt, D. Vollmer, Direct observation of drops on slippery lubricant-infused surfaces. *Soft Matter* **11**, 7617–7626 (2015).

34. O. Bliznyuk, E. Vereshchagina, E. S. Kooij, B. Poelsema, Scaling of anisotropic droplet shapes on chemically stripe-patterned surfaces. *Phys. Rev. E* **79**, 041601 (2009).

35. H. Kusumaatmaja, R. J. Vrancken, C. W. M. Bastiaansen, J. M. Yeomans, Anisotropic drop morphologies on corrugated surfaces. *Langmuir* **24**, 7299–7308 (2008).

36. H. Wijshoff, Drop dynamics in the inkjet printing process. *Curr. Opin. Colloid Interface Sci.* **36**, 20–27 (2018).

37. M. A. Hack, M. Costalonga, T. Segers, S. Karpitschka, H. Wijshoff, J. H. Snoeijer, Printing wet-on-wet: Attraction and repulsion of drops on a viscous film. *Appl. Phys. Lett.* **113**, 183701 (2018).

38. J. H. Guan, É. Ruiz-Gutiérrez, B. B. Xu, D. Wood, G. McHale, R. Ledesma-Aguilar, G. G. Wells, Drop transport and positioning on lubricant-impregnated surfaces. *Soft Matter* **13**, 3404–3410 (2017).

39. J. T. Luo, N. R. Geraldi, J. H. Guan, G. McHale, G. G. Wells, Y. Q. Fu, Slippery liquid-infused porous surfaces and droplet transportation by surface acoustic waves. *Phys. Rev. Appl.* **7**, 014017 (2017).

40. F. Boyer, C. Lapuerta, Study of a three component Cahn-Hilliard flow model. *Math. Model. Anal.* **40**, 653–687 (2006).

41. A. Daerr, A. Mogne, Pendentdrop: An ImageJ plugin to measure the surface tension from an image of a pendent drop. *J. Open Res. Softw.* **4**, e3 (2016).

42. J. H. Guan, G. G. Wells, B. B. Xu, G. McHale, D. Wood, J. Martin, S. Stuart–Cole, Evaporation of sessile droplets on slippery liquid-infused porous surfaces (SLIPS). *Langmuir* **31**, 11781–11789 (2015).
